# Measuring time in buprenorphine treatment stages among people with HIV and opioid use disorder by retention definition and its association with cocaine and hazardous alcohol use

**DOI:** 10.1186/s13722-023-00408-8

**Published:** 2023-09-02

**Authors:** Jarratt D. Pytell, Anthony T. Fojo, Jeanne C. Keruly, LaQuita N. Snow, Oluwaseun Falade-Nwulia, Richard D. Moore, Geetanjali Chander, Catherine R. Lesko

**Affiliations:** 1 Division of General Internal Medicine, Department of Medicine, University of Colorado School of Medicine, Mail Stop B180, 12631 E. 17Th Ave, Aurora, CO 80045 USA; 2 Division of General Internal Medicine, Johns Hopkins School of Medicine, Baltimore, MD USA; 3 Division of Infectious Diseases, Johns Hopkins University School of Medicine, Baltimore, MD USA; 4 Division of General Internal Medicine, University of Washington School of Medicine, Seattle, WA USA; 5 Department of Epidemiology, Johns Hopkins Bloomberg School of Public Health, Baltimore, MD USA

**Keywords:** Opioid use disorder, Retention, Polysubstance use, Buprenorphine

## Abstract

**Background:**

We use a novel, longitudinal approach to describe average time spent in opioid use disorder (OUD) cascade of care stages for people with HIV (PWH) and with OUD, incorporating four definitions of treatment retention. Using this approach, we describe the impact of cocaine or hazardous alcohol use on time spent retained on buprenorphine.

**Methods:**

We followed PWH with OUD enrolled in the Johns Hopkins HIV Clinical Cohort from their first buprenorphine treatment episode between 2013 and 2020. We estimated 4-year restricted mean time spent on buprenorphine below buprenorphine retention threshold, on buprenorphine above retention threshold, off buprenorphine and in HIV care, loss to follow-up, and death. Retention definitions were based on retention threshold (180 vs 90 days) and allowable treatment gap (7 vs 30 days). Differences in 2-year restricted mean time spent retained on buprenorphine were estimated for patients with and without cocaine or hazardous alcohol use.

**Results:**

The study sample (N = 179) was 63% male, 82% non-Hispanic Black, and mean age was 53 (SD 8) years. Patients spent on average 13.9 months (95% CI 11.4, 16.4) on buprenorphine over 4 years. There were differences in time spent retained on buprenorphine based on the retention definition, ranging from 6.5 months (95% CI 4.6, 8.5) to 9.6 months (95% CI 7.4, 11.8). Patients with cocaine use spent fewer months retained on buprenorphine. There were no differences for patients with hazardous alcohol use.

**Conclusions:**

PWH with OUD spend relatively little time receiving buprenorphine in their HIV primary care clinic. Concurrent cocaine use at buprenorphine initiation negatively impact time on buprenorphine.

**Supplementary Information:**

The online version contains supplementary material available at 10.1186/s13722-023-00408-8.

## Introduction

Williams et al. [1, 2] described eight stages of the opioid use disorder (OUD) cascade of care, ranging from primary prevention (stage 0) to recovery (stage 7), to address population and individual patient needs. The stages relevant to people with OUD are (1) diagnosis of OUD, (2) linkage to care, (3) initiation of medications for OUD, (4) treatment retention, and (5) recovery. The OUD cascade of care continues to evolve. The need to refer to treatment (“link to care”) is becoming less relevant as buprenorphine, a medication for OUD, can now be prescribed by any clinician with a Drug Enforcement Agency (DEA) registration and is increasingly available on-demand in general medical and community settings.

OUD is a chronic condition often typified by periods of abstinence and relapse to opioid use. As such, patients frequently transition on and off medications for OUD, and consequently, between stages of the OUD cascade of care. This is similar to the HIV care continuum where people with HIV (PWH) can cycle on and off antiretroviral medications and between having detectable or nondetectable HIV viral loads. Lesko et al. [3] proposed a novel method to measure and visualize the HIV care continuum by accounting for patients’ transitions between stages over time [3]. This approach could be applicable to the OUD cascade of care by accounting for patients’ transitions between OUD treatment stages and describe how patients’ time is distributed across the various stages of the OUD cascade of care. Additionally, this method can be used to estimate the impact of other factors on total time in a given treatment stage. For example, studies consistently demonstrate that cocaine and methamphetamine use have a negative impact on the length of OUD treatment episodes, whereas studies on the impact of alcohol use are mixed [4, 5].

Retaining patients on medication for OUD is necessary to reduce the adverse effects of opioid use including overdose and death [ 6–8], but there are various definitions of retention. The American Society of Addiction Medicine’s (ASAM) OUD treatment guidelines state that patients should be on OUD treatment for > 90 days for positive long-term outcomes and the National Quality Forum (NQF) set a quality metric for patients who initiate OUD treatment of 180-day retention with no gaps > 7 days [9–11].

Herein, we applied this novel, longitudinal method of describing time in treatment stages to PWH and OUD. We focused on PWH since treating OUD is critical to reaching the “Ending the HIV Epidemic” goals [12, 13] and medications for OUD decreases opioid use, mortality, and HIV transmission in this population [14–17]. We aimed to describe overall time spent on buprenorphine, time spent on buprenorphine above the retention threshold, and how estimates of time spent retained on buprenorphine differ based on the definition of retention. Lastly, we described the association between co-occurring hazardous alcohol use or cocaine use at buprenorphine initiation and time spent retained on a medication for OUD.

## Methods

### Study sample

The Johns Hopkins HIV Clinical Cohort (JHHCC) consists of PWH ages 18 years and older who are receiving HIV primary care at the John G Bartlett Specialty Practice (“Bartlett clinic”) in Baltimore, Maryland, who consent to share their data [18]. In 2013, the Bartlett clinic started office-based buprenorphine treatment for patients with OUD where buprenorphine could be initiated on-demand. The current study is a retrospective cohort analysis of all patients in the JHHCC with an OUD diagnosis who received their first buprenorphine prescription in the Bartlett clinic between January 1st 2013 and December 31st 2020 (to ensure patients had at least 1-year of follow-up). Each patient was followed from the start of their first buprenorphine prescription in the clinic in the study period until the first occurrence of the following events: censoring at the end of the observation period on December 31st, 2021, after 4 years of follow-up, or death. We administratively censored time at 4 years to ensure there were enough patients under follow-up to provide stable inference. This study was approved by a Johns Hopkins Institutional Review Board.

### Outcome measurement

**Fig. 1:**
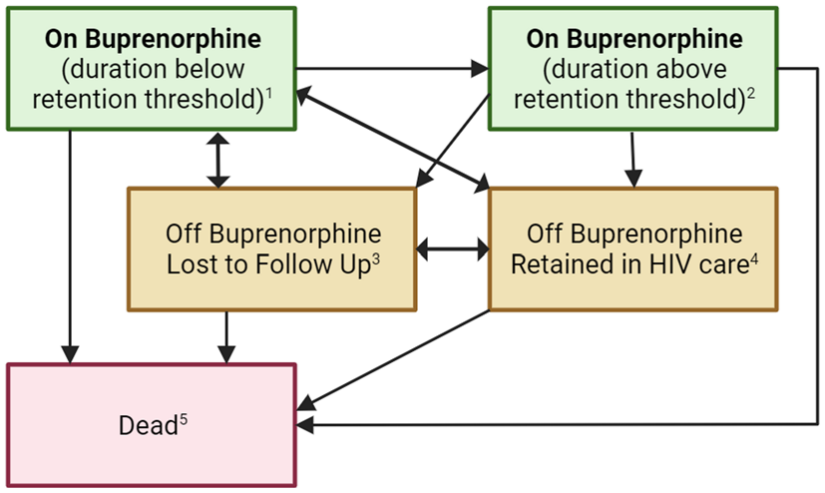
Depiction of buprenorphine treatment stages and direction of possible transitions. By definition, all participants start follow up when they initiate buprenorphine and are therefore in box 1. If the treatment episode duration is above the retention threshold (e.g., 90 or 180 days), they move to box 2. If they are in box 1 or 2 and stop buprenorphine and are lost to follow up, they move to box 3. If they are in box 1 or 2 and stop buprenorphine and are retained in HIV clinic they are in box 4. Participants can move between being lost to follow up (box 3) and retained in care (4). If after stopping buprenorphine (box 3 or 4) they restart buprenorphine they would return to box 1. Death (box 5) is a competing (terminal) event

We focused on the following buprenorphine treatment stages as shown in Fig. 1: on buprenorphine, which was further categorized into current treatment episode durations (1) below and (2) above retention thresholds; (3) off buprenorphine and lost to follow-up (LTFU); (4) off buprenorphine and retained in HIV care at the Bartlett Clinic; and (5) death. Time spent “retained” on buprenorphine only accrued after a particular treatment episode surpassed the retention threshold. For example, for a 90-day retention threshold, if a buprenorphine treatment episode was 98 days, the first 90 days would be counted in stage (1) and the last 8 days would be counted in stage (2) and we would say the patient spent 8 days retained on buprenorphine. For a 180-day retention threshold, all 98 days would be accounted in stage (1) and we would say the patient spent 0 days retained on buprenorphine. A participant’s “initial” buprenorphine prescription was their first prescription in the Bartlett clinic and not necessarily their first lifetime treatment attempt. Consistent with national quality metric measurements, patients who received an early refill were assumed to finish their existing supply before starting the new prescription [19]. Prescriptions received on the same day were assumed to be taken concurrently (e.g. buprenorphine 8mg X 14 days and buprenorphine 12mg X 14 days duration was counted as 14 covered days). We only counted buprenorphine prescriptions that were dispensed as sublingual formulations to restrict ourselves to buprenorphine for the treatment of OUD and not for pain management [9]. Data on buprenorphine prescriptions that originated outside of the Bartlett clinic and Johns Hopkins Health system were not available for research unless a patient reported the prescription to their HIV care team and the care team recorded the prescription in the patient’s medical record. Methadone is another medication for OUD and is only available in federally licensed opioid treatment programs. Federal regulations limit opioid treatment program data sharing and the JHHCC cannot capture methadone treatment administrative data including start dates, dosages, or end dates.

Lost to follow-up (LTFU) was defined as a ≥ 12-month gap between clinical visits or buprenorphine prescriptions. Patients were classified as returned to care after being LTFU if they attended a clinical visit or received a buprenorphine prescription from the clinic. Patient death dates were ascertained from clinic sources (e.g., emergency contacts or checks against the local health information exchange) and matches against the Social Security Death Index and National Death Index.

#### Defining treatment episodes and retention duration thresholds

A buprenorphine treatment episode was defined as the continuous period from an initial prescription start date to either a gap in buprenorphine prescriptions, or the end of the last prescription, whichever came first [10]. We evaluated four treatment retention definitions, ordered from the most strict to least strict criteria for retention. First, 180 days of continuous treatment without a > 7-day prescription gap based on the NQF definition [10, 19]. Second, 180 days of continuous treatment without a > 30-day prescription gap. The 30-day gap to define a new treatment episode has been used previously to evaluate buprenorphine treatment durations [20]. Third, 90 days of continuous treatment without a > 7-day gap. Fourth, 90 days of continuous treatment without a > 30-day gap. The 90-day threshold was based off the ASAM treatment guidelines which suggest that 90 days of continuous treatment is associated with improved outcomes [9]. The ASAM guidelines do not describe the gap in prescriptions that would indicate a new treatment episode.

### Covariates

#### Recent hazardous alcohol use and cocaine use

We explored whether hazardous alcohol and cocaine use at the initial buprenorphine prescription was associated with time spent retained on buprenorphine for the 2 years after initiating buprenorphine. We focused on the 2-year period after initiation because hazardous alcohol or cocaine use at initiation is more likely to affect the outcomes closer to initial buprenorphine prescription. We focused on hazardous alcohol and cocaine use for this demonstration because of the high prevalence of use of these substances in the cohort and because they were measured with good accuracy in our cohort [21, 22]. Consistent with patterns of drug use in Baltimore, there was very low (< 1%) prevalence of methamphetamine use in our cohort, so we did not consider it in this analysis. Patients were classified as having recent cocaine or hazardous alcohol use if they self-reported substance use or had substance use indicated on chart abstraction in the period 9 months before to 3 months after the initial buprenorphine prescription in the clinic. Briefly, chart abstractions are conducted for every contiguous 6-month period that patients are in care, with trained staff reviewing clinical notes to identify evidence of recent substance use (e.g., notes in a problem list). Patients self-report substance use approximately every 6 months on tablet computers in conjunction with a clinic visit. Patients report past 3-month use (yes or no) of cocaine on the Alcohol, Smoking and Substance Involvement Screening Test (ASSIST) and past 12-month alcohol use on the Alcohol Use Disorders Identification Test-Consumption (AUDIT-C) [23, 24]. Hazardous alcohol use was defined as an AUDIT-C score of ≥ 3 for women and ≥ 4 for men, where sex at birth was used to determine the cut-off. Combing chart abstraction and patient self-report improved the detection of substance use compared to using either method in isolation [21].

#### Baseline demographic and clinical covariates

We described the cohort based on demographic and clinical characteristics at the start of analytic follow-up. Gender, race, ethnicity, birth year, and HIV acquisition risk factor were collected upon enrollment into the JHHCC. Patients self-reported the most likely source of their HIV infection at enrollment into HIV care. Risk factors include injection drug use, being a man who had sex with men, or heterosexual sex. Risk factors are not mutually exclusive and may not reflect ongoing risk behaviors.

### Statistical analyses

Our analytic approach replicates approaches to visualize time in different HIV care continuum stages [3, 25, 26]. Briefly, to estimate the time in each stage, we first calculated the cumulative incidence of each instance of the following events, where the time origin is the initial buprenorphine prescription:

A. Stopping buprenorphine when the treatment episode was below the retention duration threshold (i.e., 90 or 180 days)
B. Buprenorphine treatment episode crosses the retention duration threshold
C. Stopping buprenorphine when treatment episode was greater than or equal to the retention duration threshold
D. Re-starting buprenorphine after stopping
E. LTFU after buprenorphine initiation
F. No longer LTFU after buprenorphine initiation
G. Death after buprenorphine initiation

By design all participants entered the study on buprenorphine. Participants could experience multiple instances of each of the above events with the exception of death. We estimated cumulative incidence functions separately for each instance of each event (e.g., the 3rd instance of re-starting buprenorphine after stopping). We used the Aalen-Johansen estimator to account for death as a competing event when calculating risk of events A–F [27]. The proportion of the study population in each stage at each time *t* was calculated by adding and subtracting the cumulative incidence functions at each time *t* as follows:

1. Dead after buprenorphine = cumulative incidence of death after buprenorphine initiation
2. On buprenorphine with treatment duration below retention threshold = 1 minus the sum of cumulative incidence functions for time to stopping buprenorphine treatment when episode duration is below the retention duration threshold plus sum of cumulative incidence functions for re-starting buprenorphine.
3. On buprenorphine with treatment duration above retention threshold = sum of cumulative incidence functions for time to buprenorphine treatment episode when duration above the retention threshold duration minus the sum of cumulative incidence functions for time to stopping buprenorphine when treatment episode is equal to or above the retention duration threshold.
4. Off buprenorphine and LTFU = sum of cumulative incidence of time to LTFU after buprenorphine initiation minus the sum of cumulative incidence of no longer LTFU
5. Off buprenorphine and retained in HIV care = 1 minus the proportion in stages 1–4 in this list.
6. On buprenorphine of any duration = sum of on buprenorphine below the retention duration threshold (2) and on buprenorphine with treatment duration equal to or greater than the retention duration threshold (3).

The sum of the proportions of the population in stages 1–5 is 1 and proportions can be presented as a set of stacked curves representing the distribution of the cohort across the stages over time. The area under each curve graphed separately corresponds to the restricted mean time spent in each buprenorphine treatment stage over the 4 years of follow-up after an initial buprenorphine prescription.

In secondary analyses, we compared the time spent retained on buprenorphine restricted to the 2 years of follow-up after an initial buprenorphine prescription, stratified by recent hazardous alcohol use or cocaine use at the time of the initial buprenorphine treatment episode. Because our goal with this analysis is descriptive, we present unadjusted differences [28, 29]. To handle missing data on recent hazardous alcohol and cocaine use at the initial buprenorphine initiation, we assumed data were missing at random and used a method described by Schomaker & Heumann (2018) combining bootstrap estimation with multiple imputation [30–32]. Each bootstrap sample was used to create 40 complete imputed datasets, an optimal number of imputations based on preliminary analysis [33]. To estimate 95% confidence intervals (CI) for estimates of the restricted mean time and restricted mean time differences, we took the 2.5th and 97.5th percentiles of estimates from 2000 bootstrap resamples of the data. The outcome of interest was calculated for each of the imputed dataset and the mean was calculated to give a single estimate for each bootstrap sample. This process was repeated for each of the four retention definitions. All analyses were conducted in R [34, 35].

## Results

### Study sample

Between January 1st 2013 and December 31st 2020, there were 179 participants who initiated buprenorphine treatment. Among these participants, 18% (n = 33) died during follow-up. There were 63% (n = 112) participants who identified as male and 82% (n = 147) who identified as non-Hispanic Black. Mean age at the start of the initial buprenorphine prescription was 52.8 (SD 8.0) years (Table 1). Among the participants, 56% (n = 101) reported injection drug use and 68% (n = 122) reported heterosexual sex as their possible routes of HIV transmission. A > 7-day gap (retention definitions 1 and 3) in prescriptions denoting a new treatment episode resulted in 410 buprenorphine treatment episodes while a > 30-day gap (retention definitions 2 and 4) resulted in 325 buprenorphine treatment episodes. The percentage of episodes where the duration met or exceeded the retention definition was 24% (n = 99), 32% (n = 104), 39% (n = 160), and 48% (n = 157) for retention definitions 1 through 4, respectively. Table 2 shows the number of buprenorphine treatment episodes per patient, duration, and gap between the end of one treatment episode and the beginning of the next episode, if a subsequent episode was observed.

**Table 1:** Demographic and clinical characteristics of participants with HIV and opioid use disorder who initiated clinic-based buprenorphine in the Johns Hopkins HIV Clinical Cohort, 2013–2020

	N = 179
Age^a^	52.8 (8.04)
Present gender, male	112 (63%)
Race/Ethnicity	
Non-Hispanic White	28 (16%)
Non-Hispanic Black	147 (82%)
Hispanic	3 (2%)
Other	1 (1%)
HIV Acquisition Risk Factor^b^	
Men who have sex with men	16 (9%)
Injection drug use	101 (56%)
Heterosexual sex	122 (68%)
Buprenorphine start year	
2013	40 (22%)
2014	25 (14%)
2015	3 (2%)
2016	17 (10%)
2017	19 (11%)
2018	20 (11%)
2019	30 (17%)
2020	25 (14%)
Recent cocaine use	57 (32%)
Missing	17 (10%)
Recent hazardous alcohol use	39 (22%)
Missing	14 (8%)
Died	33 (18%)

^a^ Mean (SD)

^b^ HIV acquisition risk factors are not mutually exclusive. Patients could report one or more risk factors

**Table 2:** Buprenorphine treatment episode characteristics for >7-day and >30-day prescription gap thresholds indicating a new treatment episode

	> 7-day gap	> 30-day gap
Total treatment episodes	410	325
Treatment episodes per patient, median (IQR)	2 (1,3)	1 (1,2)
Treatment episodes per patient, maximum	13	6
Treatment episode duration, median (IQR)	62 days (28, 163)	84 days, (29, 274)
Treatment episode gap duration, median (IQR)	76 days (22,311)	195 days (84, 370)

### Time spent in treatment stages

Over course of 4 years after initiation, the mean time on buprenorphine was 13.9 months (95% CI 11.4, 16.4), which represents 29% (95% CI 24%, 34%) of follow-up time. Participants spent 18.9 months (95% CI 15.6, 21.7), or 39% (95% CI 33%, 45%) of the time, off buprenorphine and retained in HIV care and 11.2 months (95% CI 8.5, 14.1), or 23% (95% CI 18%, 29%) of the time, off buprenorphine and lost to clinic follow-up. Participants who died contributed on average 4.2 months (95% CI 2.5, 6.3), or 9% (5%, 13%) of their follow-up time, in this state.

Table 3 presents the amount of time spent on buprenorphine above the retention thresholds. Retention definition 1 (≥ 180 days continuous treatment without any gaps > 7 days) was the most strict retention definition and resulted in an average of 6.5 months (95% CI 4.6, 8.5) spent retained on buprenorphine. Retention definition 4 (≥ 90 days of continuous treatment without any gaps > 30 days) was the least strict retention definition and resulted in an average of 9.6 months (95% CI 7.4, 11.8) spent retained on buprenorphine. Table 4 presents the pairwise differences in average time spent retained between retention definitions. Consider time spent retained for retention definitions 1 and 3, which both use a > 7-day gap to define the end of a treatment episode. The estimated difference in average time spent retained on buprenorphine between retention definition 1 and retention definition 3 is 1.9 (95% CI 1.2, 2.8) months. Another interpretation is that if all treatment episodes were > 180 days, the expected difference between time spent retained for definitions 1 and 3 would be 3 months (90 days). Since we observed less of a difference, it shows that patients who make it to the 90-day threshold, on average have an additional 1.9 months (95% CI 1.2, 2.8) on treatment before their treatment episode ends, or about 1.1 (95% CI 0.2, 1.8) months short of the 180-day threshold.

**Table 3:** Average number of months and percent of time spent on buprenorphine above and below retention thresholds based on retention definitions each opioid use disorder treatment stage restricted to 4 years after initiating buprenorphine treatment among patients with HIV and opioid use disorder by retention definition in the Johns Hopkins HIV Clinical Cohort, 2013–2020

Retention definition	On Buprenorphine below retention threshold		On buprenorphine at/above retention threshold	
	Months	Percent	Months	Percent
1. ≥ 180 days of treatment without > 7-day gap	7.5 (6.1, 8.9)	16 (13, 19)	6.5 (4.6, 8.5)	13 (10, 18)
2. ≥ 180 days of treatment without > 30-day gap	6.8 (5.6, 8.2)	14 (12, 17)	7.3 (5.3, 9.4)	15 (11, 20)
3. ≥ 90 days of treatment without > 7-day gap	5.3 (4.1, 6.6)	11 (9, 14)	8.4 (6.2, 10.7)	18 (13, 22)
4. ≥ 90 days of treatment without > 30-day gap	4.4 (3.5, 5.4)	9 (7, 11)	9.6 (7.4, 11.8)	20 (15, 25)

**Table 4:** Difference in estimated average number of months spent retained on buprenorphine between each of the four retention definitions

	Retention 1	Retention 2	Retention 3	Retention 4
Retention 1 ≥ *180 days of treatment without* > *7-day gap*	0	0.8 (0.3,1.4)	1.9 (1.2, 2.8)	3.1 (2.1, 4.2)
Retention 2 ≥ *180 days of treatment without* > *30-day gap*		0	1.1 (0.4, 1.8)	2.3 (1.4, 3.2)
Retention 3 ≥ *90 days of treatment without* > *7-day gap*			0	1.2 (0.5, 2.1)
Retention 4 ≥ *90 days of treatment without* > *30-day gap*				0

### Visualization of time spent in treatment stages

**Fig. 2:**
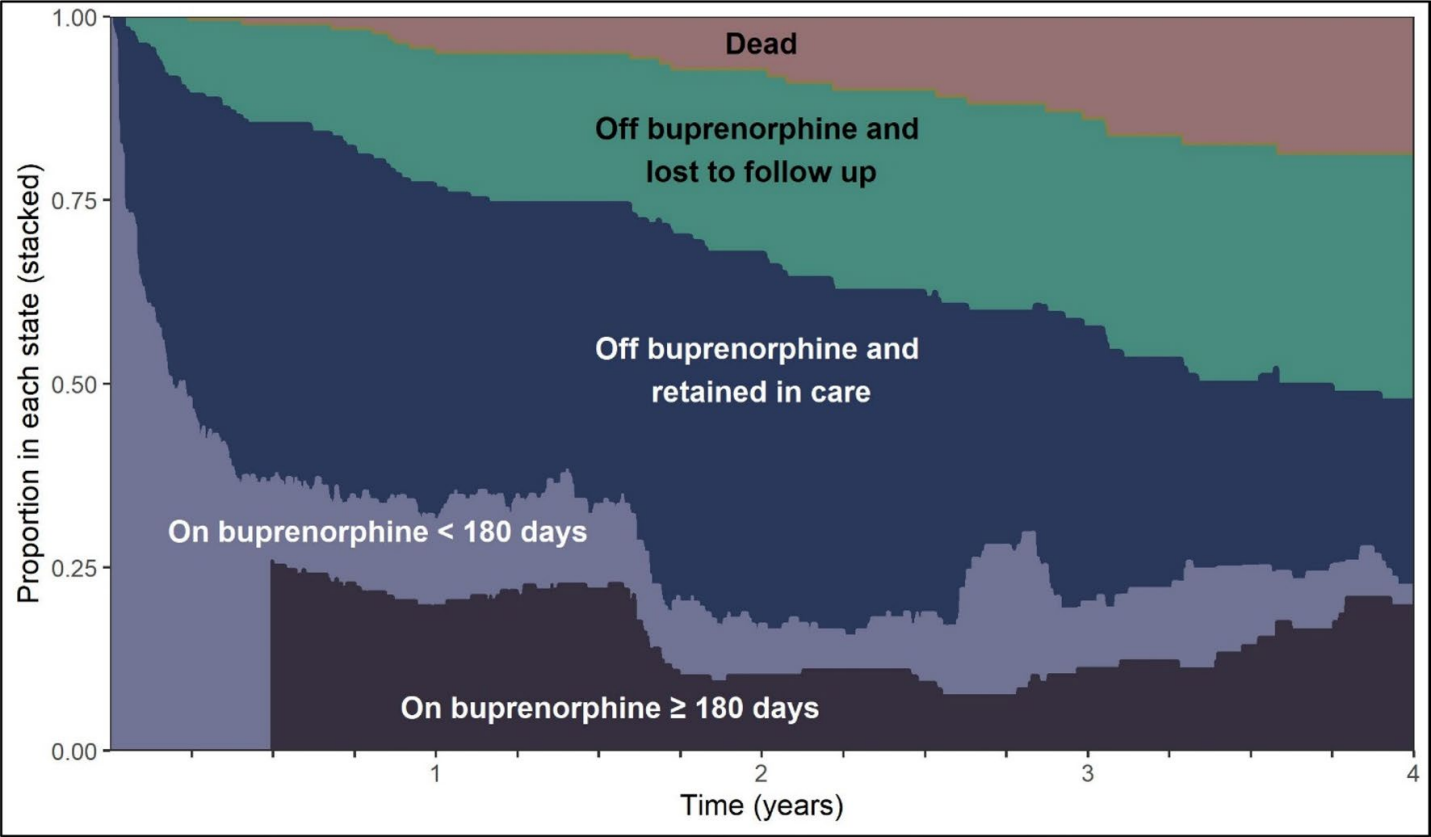
Proportion of participants with HIV and opioid use disorder who initiated clinic-based buprenorphine in each opioid use disorder treatment stage in the Johns Hopkins HIV Clinical Cohort restricted to 4-year follow-up, with retention defined as ≥ 180 days of treatment without a > 7-day gap

Figure 2 depicts these data for retention definition 1. The area between curves is the total amount of time that an individual spends in each stage on average. The interpretation at a given time point is the probability of a person being in each stage. For example, at 180 days after initiating buprenorphine treatment, the probability of a patient having been on buprenorphine for ≥ 180 days is 25% (dark purple). At 1.75 years, the probability of a patient being on buprenorphine of any treatment episode length decreases to 20% (light purple), which steadily increases to 25% by year 4. Additional file 1: Fig. S1 presents the stacked proportions for all retention definitions and shows, across all definitions of retention, in years 1–4, fewer than 25% of patients are on buprenorphine and retained.

### Time spent retained in treatment, stratified by cocaine or hazardous alcohol use

Out of 179 participants, 32% (n = 57) had recent cocaine use and 22% (n = 39) of participants had recent hazardous alcohol use (Table 1). Data were missing on recent cocaine or hazardous alcohol use for 10% (n = 17) and 8% (n = 14) of participants, respectively.

Table 5 shows the average time spent retained on buprenorphine over 2 years after initiation for each of the retention definitions overall and stratified by recent cocaine or hazardous alcohol use. Similar to the results discussed above, the average time retained on buprenorphine over 2 years after initiation was lowest when using the most strict retention definition 1 (≥ 180 days of treatment without > 7-day gap; 6.4 months [95% CI, 4.1, 8.9]) and highest for the least strict definition 4 (≥ 90 days of treatment without > 30-day gap; 10.4 months [95% CI 7.5, 13.7]).

**Table 5:** Average number of months spent retained on buprenorphine above the retention thresholds overall and by the presence of cocaine or hazardous alcohol use at initiation restricted to 2 years after initiating buprenorphine treatment among participants with HIV and opioid use disorder in the Johns Hopkins HIV Clinical Cohort, 2013-2020

Retention definition	Overall	Cocaine use			Hazardous alcohol use		
		No	Yes	Difference	No	Yes	Difference
Retention 1 ≥ *180 days of treatment without* > *7-day gap*	6.4 (4.1, 8.9)	4.1 (3.0, 5.2)	2.3 (1.2, 3.8)	1.8 (0.1, 3.5)	3.7 (2.7, 4.8)	2.8 (1.3, 4.5)	0.9 (–1.0, 2.8)
Retention 2 ≥ *180 days of treatment without* > *30-day gap*	7.5 (5.1, 10.2)	5.0 (3.8, 6.3)	2.5 (1.3, 3.9)	2.5 (0.6, 4.2)	4.5 (3.4, 5.7)	3.1 (1.6, 4.9)	1.4 (–0.6, 3.4)
Retention 3 ≥ *90 days of treatment without* > *7-day gap*	9.0 (6.2, 12.1)	5.7 (4.4, 7.1)	3.3 (1.8, 5.0)	2.4 (0.4, 4.4)	5.2 (4.0, 6.5)	4.0 (2.1, 6.0)	1.2 (–1.1, 3.5)
Retention 4 ≥ *90 days of treatment without* > *30-day gap*	10.4 (7.5, 13.7)	6.8 (5.4, 8.4)	3.6 (2.1, 5.3)	3.2(1.1, 5.3)	6.1 (4.9, 7.5)	4.4 (2.5, 6.6)	1.7 (–0.7, 4.1)

The separate models for recent cocaine use and hazardous alcohol use applied multiple imputation and bootstrap estimation techniques (refer to Methods section 2.4 for more detail). The "Overall" column presents combined estimations from the models evaluating differences in cocaine use. Hence, the sum of the "No" and "Yes" columns in the cocaine use category equates to the value in the "Overall" column. Small discrepancies (1–2%) may be observed between the "Overall" column and the summed values of the "No" and "Yes" columns in the hazardous alcohol use category. These slight variations are anticipated outcomes of bootstrap estimation

For retention definition 1 (≥ 180 days of treatment without a > 7-day gap), recent cocaine use was associated with 1.8 (95% CI, 0.1, 3.5) fewer months spent retained on buprenorphine over 2 years after first buprenorphine initiation. Hazardous alcohol use was associated with 0.9 (95% CI − 1.0, 2.8) fewer months spent retained on buprenorphine over 2 years after initiation. For all retention definitions, cocaine use was associated with a significant reduction in the average time spent on buprenorphine over 2 years after buprenorphine initiation. No statistically significant differences were observed for hazardous alcohol use across all definitions.

## Discussion

Applying a novel method accounting for multiple treatment episodes and transitions between treatment stages in a retrospective study of 179 PWH initiating buprenorphine for the treatment of OUD in an office-based setting, integrated within a HIV specialty clinic, we found very low probability of being retained on buprenorphine across 4 years of follow-up. In addition, consistent with a prior study, we found that the definition of retention directed impacted the estimates of number of treatment episodes, treatment episode durations, and time spent on buprenorphine [36]. These results highlight the need to understand not only the barriers to retention in OUD care cascade but also how the definition of retention influences our conclusions.

Two factors constituted each retention definition. First, the threshold for what is considered retained and second, the gap between prescriptions that would constitute a new treatment episode. There is limited research on the optimal length of buprenorphine treatment. A simulation study based on a cohort of patients with OUD in the United States Veterans Health Administration suggests 2 months of buprenorphine treatment and 4 months of methadone was needed to reduce all-cause mortality [11]. While reducing mortality is a very important goal, recovery from OUD is the final stage of the OUD cascade of care and people might require longer treatment durations to attain recovery. The National Institute of Drug Abuse’s (NIDA) Principles of Drug Addiction [37] states that most people being treated for a substance use disorder need 3 months in treatment to reduce drug use and achieve optimal outcomes. For this reason, the ASAM treatment guidelines state that there is limited effectiveness of treatment durations less than 90 days, but state that there is no recommended time limit for pharmacological treatment [9]. Although the NQF committee identified a duration of 180 days for retention, it did not recommend this measure be used in pay-for-performance due to our limited understanding of optimal treatment durations. A NIDA Clinical Trails Network study “Optimizing Retention, Duration, and Discontinuation Strategies for Opioid Use Disorder Pharmacotherapy” (CTN-0100) is ongoing to explore the effect of treatment duration on post-discontinuation outcomes [38].

Second, retention depends on the gap in treatment that defines a new treatment episode. The NQF committee specifies a > 7-day gap in treatment in their performance measure, while the ASAM and NIDA reports do not specify a gap. From a pharmacologic perspective, buprenorphine can suppress cravings for up to 2 days after last dose [39] and patients would likely need to undergo re-induction after this period, particularly if they have been using other opioids like heroin or fentanyl. Practically, many patients use buprenorphine obtained from friends, family, the illicit market, or left over from previous prescriptions in a self-directed manner when there are gaps in their prescriptions [40–42]. The relatively large difference in number of treatment episodes based on a 7- or 30-day gap in prescriptions suggest that patients often have gaps in prescriptions. Numerous studies describe the multi-level barriers to OUD treatment for PWH [43–45] and patients are often report ambivalence about medications for OUD, largely due to the associated stigma and perceptions about what it means to be “in recovery” [46, 47]. It is important to note that the present analysis is based on buprenorphine prescriptions, not dispensed medication, and there could be delays in filling medications relative to the prescription date, which would lead us to underestimate the actual gap in treatment and overestimate treatment duration and retention.

These factors aside, we found that regardless of the retention definition, the average amount of time an individual participant spent on buprenorphine and retained in this clinic was low—13.9 months over the 4 years after buprenorphine initiation. Based on visual inspection of the graphs, the probability that a patient will be on buprenorphine at a given point in the 1.5 to 4 years after initiation is less than 25% across all retention definitions suggesting that long-term retention on buprenorphine originating from the clinic is low in this cohort. This finding is consistent with results from similar office-based settings. In a single-site, primary care office-based opioid treatment clinic in New York City from 2006 to 2013, 1-year buprenorphine treatment retention was about 20% [48]. Earlier studies of primary-care office based opioid treatment had 1-year retention rates as high as 50% [49, 50]. A large sample of 1237 patients at a safety-net office-based opioid treatment program found that 45% of patients were retained for ≥ 1 year. Our results continue to highlight the need to develop and test interventions to improve patient retention on buprenorphine in outpatient settings [51].

We sought to demonstrate a potential application of this method by describing the association between cocaine or hazardous alcohol use and time spent retained on buprenorphine. While results should be considered exploratory, we observed significantly less time retained on buprenorphine among participants with cocaine use, consistent with previous research [4, 5]. Notably, differences were found for all retention definitions suggesting this finding does not depend on the retention definition. Hazardous alcohol use at buprenorphine initiation did not result in statistically significant differences in a time spent retained on buprenorphine. Multiple substance use is a common clinical situation and these results show that different patterns of substance use likely impact buprenorphine retention in different ways [52, 53]. It is possible that patients with cocaine or hazardous alcohol use might be referred more often to higher levels of care (e.g., specialty outpatient or inpatient addiction treatment settings). Given that these treatment episodes are not documented in our data, patients referred to a higher level of care might continue buprenorphine treatment outside the clinic. This could lead to a misclassification in our analysis, implying they are off buprenorphine. Such misclassification could result in overestimating retention differences when compared to patients without cocaine or hazardous alcohol use, who might be more inclined to continue treatment within the Bartlett clinic.

This study is limited in that only buprenorphine prescriptions that were prescribed or recorded by clinicians in the Bartlett clinic were included. Patients could receive buprenorphine through other clinics or federally licensed opioid treatment programs. However, the Bartlett clinic has characteristics of low-threshold treatment, including treatment on demand, and patients who remain active in HIV care in the clinic would have the opportunity to receive buprenorphine from the clinic [54]. We suspect that patients are likely to use the Bartlett clinic as their primary source of OUD treatment with buprenorphine. Incorporating prescription drug monitoring program and opioid treatment program medication dispensing data would yield a more comprehensive picture of patient transitions between treatment settings and stages within the OUD cascade of care. Additionally, we only had data on buprenorphine prescriptions and not whether those prescriptions were dispensed or whether dispensation was delayed. Inclusion of pharmacy dispensing would improve the accuracy of our estimates. Finally, our findings are based on data from an office-based opioid treatment program in one HIV care clinic and may not be generalizable across all care settings. In particular, because Baltimore has a low prevalence of methamphetamine use, buprenorphine use patterns may be different in our clinic compared to locations with a higher prevalence of methamphetamine use.

Despite these limitations, we believe this novel approach of estimating time in OUD treatment stages could improve our understanding of OUD treatment and factors that impact retention on medications for OUD. Future applications of this approach could expand to include other clinically-relevant treatment stages including tapering, early discontinuation before meeting a retention threshold, or transitioning to a different medication for OUD (e.g., methadone). In addition, recent data suggests that Black and other minoritized communities have disproportionately high opioid overdose deaths and disparities in treatment with medications for OUD [55, 56]. Evaluating how other factors, including the local drug supply (e.g., fentanyl saturation), state policies, other common substance use (e.g., cannabis), and patient demographics impact time in treatment stages could help inform individual and public health interventions.

## Conclusion

We demonstrate how a novel approach can account for patient transitions between buprenorphine treatment stages to estimate the average time spent in each stage over time. Participants spent less than 25% of their time on buprenorphine treatment in the same clinic in the 4 years following buprenorphine initiation. For participants with recent cocaine use at initiation, less time is spent retained on buprenorphine. Our results highlight the necessity of incorporating multiple data sources, including prescription drug monitoring program and opioid treatment program data, into clinical research. Future studies that incorporate diverse, rich data sources could provide a comprehensive assessment of patients’ time in treatment stages and be used to explore optimal treatment durations and factors associated with retention on medications for OUD.

### Supplementary Information

**Additional file 1: Figure S1.** Depiction of treatment buprenorphine treatment states for each of the retention definitions.

## Data Availability

The datasets generated and/or analyzed during the current study are not publicly available due their containing information that could compromise the privacy of research participants as incorporated in the informed consent, but are available from the corresponding author on reasonable request.
