# Supplementary material for: Measuring time in buprenorphine treatment stages among people with HIV and opioid use disorder by retention definition and its association with cocaine and hazardous alcohol use
Source: Addict Sci Clin Pract. 2023 Sep 2;18:51. doi: 10.1186/s13722-023-00408-8 (PMC10474763; doi:10.1186/s13722-023-00408-8)
Supplement: Supplementary file 1 — Additional file 1: Figure S1. Depiction of treatment buprenorphine treatment states for each of the retention definitions. [file 13722_2023_408_MOESM1_ESM.docx]

Additional for “Measuring time in buprenorphine treatment stages among people with HIV and opioid use disorder by retention definition and its association with cocaine and hazardous alcohol use.”

**eFigure 1:** Depiction of treatment buprenorphine treatment stages for each of the retention definitions.


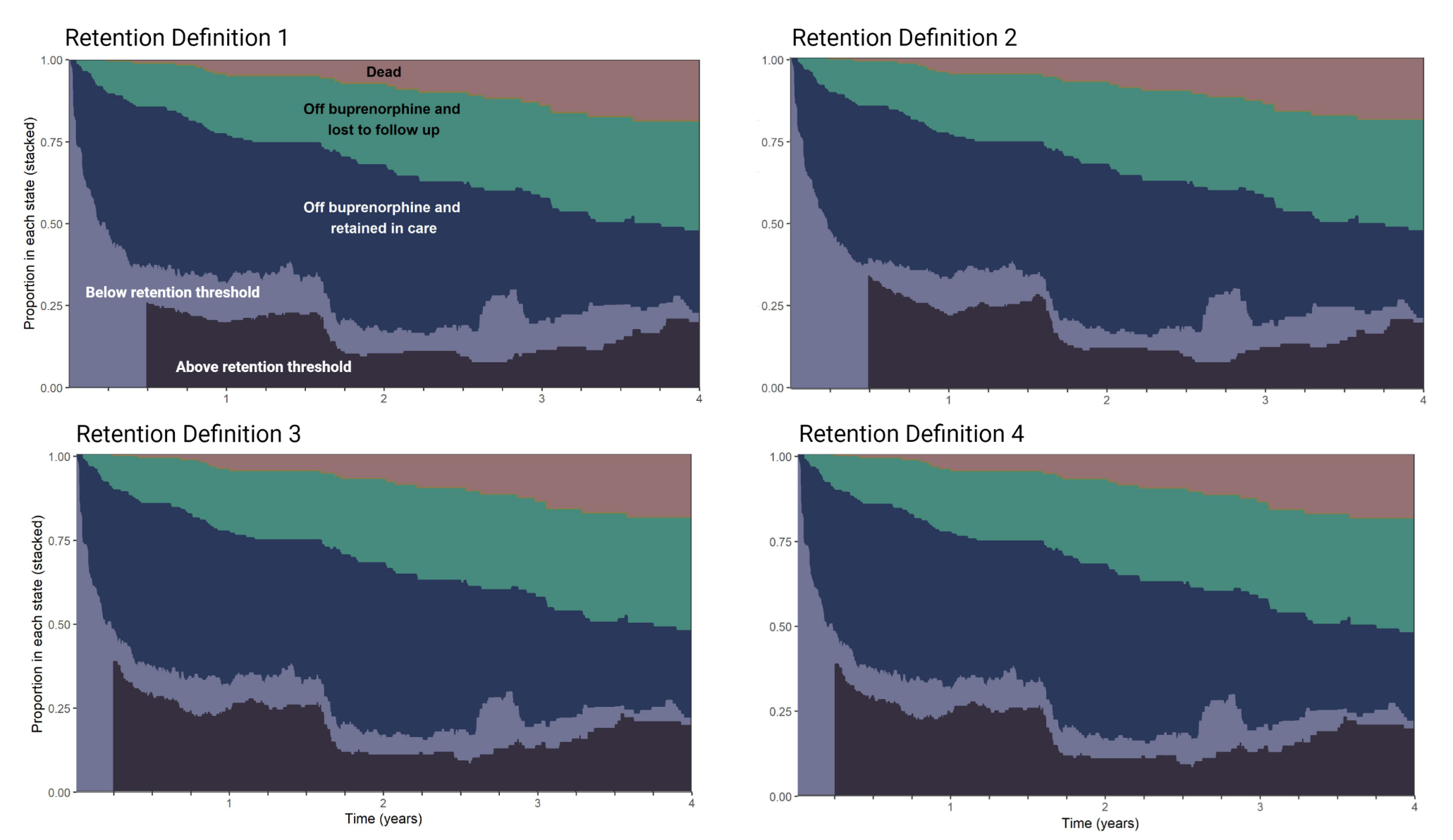


Retention 1: *≥180 days of treatment without >7-day gap;* Retention 2: *≥180 days of treatment without >30-day gap;* Retention 3: *≥90 days of treatment without >7-day gap;* Retention 4: *≥90 days of treatment without >30-day gap*
